# Pattern Recognition Approach for the Screening of Potential Adulteration of Traditional and Bourbon Barrel-Aged Maple Syrups by Spectral Fingerprinting and Classical Methods

**DOI:** 10.3390/foods11152211

**Published:** 2022-07-25

**Authors:** Kuanrong Zhu, Didem P. Aykas, Luis E. Rodriguez-Saona

**Affiliations:** 1 Department of Food Science and Technology, The Ohio State University, 110 Parker Food Science and Technology 2015 Fyffe Road, Columbus, OH 43210, USA; zhu.1421@buckeyemail.osu.edu; 2 Department of Food Engineering, Faculty of Engineering, Adnan Menderes University, Aydin 09100, Turkey; didem.cinkilic@adu.edu.tr

**Keywords:** maple syrups, adulteration, FT-IR, Raman, GC-MS, bourbon barrel aged

## Abstract

This study aims to generate predictive models based on mid-infrared and Raman spectral fingerprints to characterize unique compositional traits of traditional and bourbon barrel (BBL)-aged maple syrups, allowing for fast product authentication and detection of potential ingredient tampering. Traditional (*n* = 23) and BBL-aged (*n* = 17) maple syrup samples were provided by a local maple syrup farm, purchased from local grocery stores in Columbus, Ohio, and an online vendor. A portable FT-IR spectrometer with a triple-reflection diamond ATR and a compact benchtop Raman system (1064 nm laser) were used for spectra collection. Samples were characterized by chromatography (HPLC and GC-MS), refractometry, and Folin–Ciocalteu methods. We found the incidence of adulteration in 15% (6 out of 40) of samples that exhibited unusual sugar and/or volatile profiles. The unique spectral patterns combined with soft independent modeling of class analogy (SIMCA) identified all adulterated samples, providing a non-destructive and fast authentication of BBL and regular maple syrups and discriminated potential maple syrup adulterants. Both systems, combined with partial least squares regression (PLSR), showed good predictions for the total °Brix and sucrose contents of all samples.

## 1. Introduction

Native Americans are widely recognized as the first to discover the sweet sap dripping from the broken bark of sugar maple (Acer saccharum), which is the only ingredient of natural maple syrup products [1,2]. Maple syrup has a reputation for being a nutritious, classical sweetener and having a unique taste and flavor. According to the United States Department of Agriculture (USDA), the US production of maple syrup in 2019 totaled 4.37 million gallons with an estimated value of USD 135 million [3].

Pure maple syrups produced in North America comprise 68 ± 4% sucrose, 0.43 ± 1.11% glucose, 0.30 ± 0.54% fructose, a small amount of amino acids, various phenolic compounds, a trace amount of organic acids, including malic and fumaric acids, minerals, and salts [4]. Maple syrup is superior to other sweeteners because of its rich phenolic and phytohormone contents, which possess antioxidant properties and produce low glycemic and insulinemic responses [5].

Barrel aging is a process in which wine or spirits are stored and aged in wooden barrels. Chemical reactions take place during the aging process in which wine or spirits are absorbed into the wood constituents, including volatile compounds that contribute to the smell property and non-volatile compounds that correlate with color and mouth-feel properties [6]. In recent years, aging maple syrup in bourbon barrels has become popular and creates more value than traditional pure maple syrup. Bourbon barrel (BBL)-aged maple syrup is produced by aging traditional maple syrup in oak bourbon barrels for several weeks to months to develop richer bourbon flavor without adding any other ingredients [7]. In addition, in the process of aging, it is crucial to control the strength of extracted bourbon flavor to be neither too weak nor too strong to overshadow the maple flavor [7].

Maple syrup manufacturing is rather costly since it is regulated by law, specifying that the only ingredient in maple syrup is the maple sap [1]. Although sap contents may vary from different maple trees, in general, 1 L of traditional maple syrup is produced by concentrating around 35 L of maple sap to 66 °Brix [1]. Therefore, maple syrup could be potentially adulterated by adding inexpensive cane, beets, or corn syrup to the boiling sap or by blending the maple syrup with corn syrup due to financial incentives [1]. Since the taste of a small amount of cane sugar or corn syrup added to maple syrup is almost undetectable, the inclination to increase yields by fraudulent means can be substantial [8]. In addition, the even higher price of BBL-aged maple syrups may prompt the potential counterfeit of the aging process by having a minimum aging activity or by using an unqualified aging barrel that does not contain adequate bourbon residuals.

Traditional authentication methods, including chromatography, mass spectrometry, and stable isotope ratio analysis, have been applied to maple syrup studies [4,9]. Stuckel and Low developed a methodology to fingerprint oligosaccharides in maple syrup and to detect adulteration of high-fructose corn syrup and beet medium invert sugar via anion-exchange HPLC [10]. Carro et al. [9] authenticated maple syrup samples by using carbon stable isotope ratio analysis. An improved method of stable carbon isotope ratio mass spectroscopy was established by Tremblay and Paquin [11] with the isolation of malic acid to detect the addition of beet and cane sugar in maple syrup.

However, these methods are time-consuming and cost-prohibitive for most maple syrup manufacturers due to the requirements of expensive instrumentation and trained personnel [12]. Advances in the miniaturization of vibrational spectroscopy instruments combined with powerful chemometrics can overcome those problems by offering fast product authentication, non-destructive and real-time analysis [12]. Fourier transform infrared spectroscopy (FT-IR) is a vibrational spectroscopy technique that measures the absorbance and transmittance of infrared light. Raman spectroscopy is another type of vibrational spectroscopy using an intense light beam, such as a laser, to excite the sample molecules by inducing Raman-active vibrational modes and measuring inelastically scattered photons [13]. Since FT-IR measures the absorption of light, it is effective in measuring colored and fluorescence samples. At the same time, the presence of fluorescence creates optical noise for Raman measurements, which easily obscures the spectral fingerprint of the sample [14]. In addition, Raman scattering is based on polarizability changes in functional groups during molecule vibration [15]. Therefore, nonpolar bonds tend to give an intense Raman signal, while water in samples could be virtually disregarded due to a weak Raman signal [15]. Based on the reasons above, FT-IR and Raman are often used as complementary technologies for broader chemical identification. However, limited studies have employed vibrational spectroscopy for the authentication of maple syrups, and there is a gap in knowledge on the performance of portable/handheld devices for the detection of adulteration in maple syrups. Paradkar and others [8] reported the use of benchtop FT-IR, NIR, and FT-Raman systems to detect corn syrup adulteration in maple syrup. Mellado-Mojica and others [16] used FT-IR to contrast the carbohydrate composition of maple syrups against other sweeteners. In addition, chemometrics or multivariate analysis techniques have been proven to be successfully applied in the study of food matrices [17,18].

The objective of this research was to evaluate portable mid-infrared and Raman devices in generating predictive models for the non-destructive and fast fingerprinting of traditional and BBL maple syrups, allowing for product authentication and detection of potential ingredient tampering. This is the first study that characterizes a premium maple syrup aged in oak bourbon barrels, as there is no standard of identity or any other study reporting on this novel product. The use of miniaturized vibrational spectroscopies in maple syrup authentication can provide the industry with field-deployable devices for quality control and for preventing adulteration with cheaper ingredients.

## 2. Materials and Methods

### 2.1. Samples

Traditional and BBL maple syrup samples were kindly provided by a local maple syrup farm in Jefferson, OH (*n* = 12 (traditional), *n* = 8 (BBL)) and were purchased from local grocery stores in Columbus, OH (*n* = 7 (traditional), *n* = 5 (BBL)) that consisted of traditional maple syrups (*n* = 19), including dark, amber, and golden grades, and BBL-aged maple syrup (*n* = 13). In addition, table syrups (corn *n* = 2, cane *n* = 2, and mixture, consisting of cane, maple, and agave syrups *n* = 1) (*n* = 5) were purchased from grocery stores in Columbus, OH, USA for generating training models. An independent external validation set, consisting of traditional (*n* = 4) and BBL (*n* = 4) maple syrups, was purchased from an online vendor (Amazon.com, Inc. Seattle, WA, USA). All samples were stored in the refrigerator at 4°C and were equilibrated at room temperature before spectroscopic measurements and reference analyses.

### 2.2. Reference Analyses

#### 2.2.1. °Brix

°Brix of each sample was measured with the heat-controlled refractometer (RX 5000i ATAGO, Bellevue, WA, USA). The syrup sample (~0.3 mL) was carefully pipetted onto the prism of the refractometer without creating any air bubbles, and measurement at 22 °C was recorded.

#### 2.2.2. High-Performance Liquid Chromatography

Concentrations of sucrose, fructose, and glucose were measured with high-performance liquid chromatography (HPLC) (Shimadzu, Columbia, MD, USA). The HPLC was equipped with a SIL-20AHT autosampler, a CTO-20A oven, an LC-6AD pump, a CBM-20A controller, and a RID-10A refractive index detector. The syrup sample (~0.5 g) was weighed into a 15 mL centrifuge tube and diluted with (~7 mL) HPLC grade water. The actual weights of syrup and water were recorded. The mixture was vortexed for 40 sec and was filtered through the 0.2 µm filter (Phenomenex^®^, Torrance, CA, USA), and then filled into an HPLC vial. Isolated sugars were segregated by a Rezex RCM-Monosaccharide Ca+ 300 × 7.8 mm column (Phenomenex^®^). Sugars were eluted under the isocratic condition at 80 °C, using HPLC grade water as a mobile phase at a 1 mL/min flow rate for 20 min. LC Solutions software (Version 3.0, Shimadzu, Columbia, MD, USA) was used to integrate chromatograms automatically. The standard curve with concentration ranges from 10 to 50 mg/mL (>99% purity, Fisher Scientific, Fair Lawn, NJ, USA) was plotted to calculate each sugar content.

#### 2.2.3. Total Phenolics

Total phenolic contents of maple syrups were determined with Folin–Ciocalteu (FC) method described by Waterhouse with some modification [19]. The syrup sample (~0.8 g) was weighed into a microcentrifuge tube and diluted with deionized (DI) water (~0.4 mL). The actual weights of syrup and water were recorded, and the diluted sample was vortexed for 40 s. The diluted sample (50 µL) was pipetted into a 96-well plate, followed by 200 µL DI water and 20 µL FC reagent. The mixture was mixed thoroughly by pipetting and incubated for 7 min at room temperature. The sodium carbonate solution (100 µL) was added to the mixture and incubated for 2 h under dark conditions at room temperature. The equilibrated sample’s absorbance was measured at 765 nm. A standard curve constructed with gallic acid standard with concentration ranges from 125 to 800 µg/mL was used to quantify total phenolics. Results were expressed as micrograms of gallic acid equivalent (GAE) per 1 mL of distilled water.

#### 2.2.4. Gas Chromatography—Mass Spectrometry

The volatile composition of the samples was identified using gas chromatography–mass spectrometry (GC-MS) (Agilent 7820A GC connected to a 5977B MS, Agilent Technologies, Santa Clara, CA, USA). A total of 1 g maple syrup sample was placed into a 20 mL clear screw-tread glass headspace vial (Restek, Bellefonte, PA, USA), and the vial was sealed with an 18 mm screw-tread PTFE/silicone septa vial cap (Restek, Bellefonte, PA, USA). The vial with the sample was placed onto a heating plate at 40 °C for 30 min to equilibrate the volatile compounds. A preconditioned SPME fiber (50/30 µm DVB/CAR/PDMS coated) (Supelco, Sigma-Aldrich, Bellefonte, PA, USA) assembly was inserted in the vial through the septa of the cap, and the volatiles were trapped by the fiber for 15 min. After the trapping, fiber assembly was removed from the vial and directly inserted through the GC-MS injection port. Compounds were desorbed at 250 °C for 1 min in splitless mode, followed by a 30 s purge flow (50 mL/min) to clean the fiber. A quality control (QC) sample was prepared by pooling 100 µL of each sample to monitor the performance of the method and identify qualified peaks. 2,3-hexanedione (Sigma-Aldrich, St. Louis, MO, USA) was prepared at 10 ppm concentration with distilled water and used as an internal standard (IS) to correct the variation through the run. A 40 µL of IS solution was added to each sample. The volatile compounds were separated on a DB-Wax column (60 m × 250 μm × 0.25 μm) (Agilent Technologies, Santa Clara, CA, USA). The oven was held at 60 °C for 5 min then ramped to 130 °C at 5 °C/min. This was followed by the second ramp of 5 °C/min to 240 °C, where it was held for 8 min. The MS acquisition was performed in scan mode between masses 25–300 m/z at a 2.7 scans/s rate. Data were extracted in the Agilent Masshunter Quantitative Analysis software. The spectral background was corrected, and only peaks that had a signal-to-noise (S/N) ratio higher than the detection limit (S/N > 5) were conserved. All compounds were tentatively identified using the NIST 14.L database by a Mass Spectral Library search.

#### 2.2.5. Statistics of Reference Analysis

All reference laboratory analyses were performed in duplicate, and their range, minimum, maximum, mean, and standard deviation (SD) are determined. In addition, the standard error of laboratory (SEL) was calculated according to the method of Kovalenko et al. [20].

### 2.3. Vibrational Spectroscopy

#### 2.3.1. Mid-Infrared Analysis

The mid-infrared data were collected with portable FT-IR spectroscopy (Agilent Technologies, Santa Clara, CA, USA) attached with a triple-reflection diamond Attenuated Total Reflectance (ATR) crystal. The ATR crystal has a sampling surface of 2 mm diameter and a 200 µm active area and provides ~6 µm depth of penetration. In addition, the FT-IR system is also attached with a deuterated triglycine sulfate (DTGS) detector and a Zinc Selenide (ZnSe) beam splitter. Spectra were collected from 4000 to 700 cm^−1^ with a resolution of 4 cm^−1^. Sixty-four spectra were co-added in each sample collection to increase the signal-to-noise ratio. A spectral background was taken in between every measurement to reduce the environmental changes. Approximately 0.2 g of syrup sample was directly applied to the sampling surface of the ATR crystal, confirming that full coverage of the sample was achieved. Spectra for each sample were collected in triplicate, and collected spectral data were documented by Agilent MicroLab PC software (Agilent Technologies, Santa Clara, CA, USA).

#### 2.3.2. Raman Analysis

About 3 mL of syrup sample was filled in a quartz cuvette (Hellma Analytics, Mulheim, Germany) with a 10 mm light path and measured with a compact benchtop Raman spectrometer WP 1064 (Wasatch Photonics, Durham, NC, USA). The Raman spectrometer was coupled with a laser operating at 1064 nm and an Indium Gallium Arsenide (InGaAs) detector. Spectra were collected from 350 to 1500 cm^−1^ with a resolution of 4 cm^−1^. In addition, three scans were co-added and averaged to increase the signal-to-noise ratio of the spectrum, which has an integration time of 3 s. A spectral background was taken in between every measurement to reduce the environmental changes. The spectral collection was performed in triplication for each sample, and collected spectral data were documented by EnlightenTM software (Wasatch Photonics, Durham, NC, USA).

### 2.4. Multivariate Data Analysis

FT-IR and Raman spectra were exported and analyzed using Pirouette^®^ multi-variate statistical analysis software (version 4.5, Infometrix Inc., Bothell, WA, USA). The mean spectrum of the three replicates was used for the statistical analysis. The collected FT-IR and Raman data were preprocessed with mean-centering to reduce micro multicollinearity and transformed with the Savitzky–Golay (SG) algorithm (35-point polynomial filter) in soft independent modeling of class analogy (SIMCA) and partial least squares regression (PLSR) models [21]. The SG algorithm was used to resolve overlapping spectroscopic signals and to improve their properties, also surpassing the instrument noise [22]. A 35-point smoothing filter was found as an optimal window length for our data set. The optimum window length was chosen to resolve essential details in the collected spectra and lessen signal noise. Mean centering and SG algorithms were chosen after evaluating the preprocessing quality of the spectral data with other options, including smoothing, normalization, and divide by; however, they were all outperformed by the combination of mean-centering and SG. An additional data transformation step of normalization (2-norm × 100) was applied in the case of PLSR analysis.

#### 2.4.1. SIMCA

Classification analyses of maple syrups were performed using a supervised pattern recognition classification method SIMCA, which uses the previous understanding of the category membership of samples to classify new unrevealed samples in one of the known classes based on the pattern of measurements [23]. The cross-validation (leave-out-out) was used to assess the performance of the training model by analyzing the misclassification and generalization error [24]. The performance of the SIMCA model was also assessed with class projections, discriminating power, misclassification, and interclass distances (ICD), which interpret the quantitative similarity or dissimilarity of different classes and are widely accepted as samples that can be well differentiated when ICD >3 [24].

#### 2.4.2. PLSR

The quantitative PLSR method was used for developing predictive training models of °Brix and sucrose contents by combining features from multiple linear regression and PCA. Cross-validation (leave-one-out) was used for internal validation of the training model. All syrup samples (*n* = 37) were randomly separated into calibration (~80% of the total samples) and external validation (~20% of the total samples) sets to evaluate the robustness of the trained models. Triplications of the same sample were used either in the training set or in the external validation set. The performance of the PLSR model was assessed with a correlation coeffect of cross-validation (Rcal) and predictions (Rval), standard error of cross-validation (SECV) and predictions (SEP), outlier diagnostics, leverage, and residual analysis [25]. Samples with high residuals and leverage were re-analyzed and excluded from the model if needed.

## 3. Results and Discussion

### 3.1. Characterization of Maple Syrup Samples

Reference analysis results for total soluble solids (°Brix), sugar (sucrose, fructose, and glucose), and total phenolics for all samples, including traditional maple syrup, bourbon barrel (BBL)-aged maple syrup, and table syrups (corn, cane, and mixture—consisted of cane, maple, and agave syrups) are summarized in Table 1.

Traditional maple syrups and BBL maple syrups showed similar total soluble solids (°Brix) contents (Table 1). The °Brix values of maple syrups (65.4–68.7° with an average of 66.6 ± 0.7°) were within the range reported by Stuckel and Low (62.2–74.0° with an average of 67.0 ± 1.6°) [4] and Perkins (66–68°) [26]. Sucrose, fructose, and glucose contents of traditional and BBL maple syrups are summarized in Table 1. We found no significant difference (*p* = 0.98, *p* > 0.05) in the sugar content between traditional and BBL maple syrups. Most sucrose contents of maple syrups agree with the reported literature (51.7–75.6% with an average of 68.0 ± 4.0%) [4], while four labeled as traditional maple syrup samples were far below the range (22.0, 23.5, 36.1, and 50.6%). These same four samples have much higher fructose (9.5–17.1%) and glucose (9.7–17.1%) contents than the literature reports (fructose 0.3 ± 0.5%, and glucose 0.4 ± 1.1%) [4]. In Morselli’s study, fructose content in maple syrup was undetectable, and glucose content ranged from 0–7.3% [27]. The glucose to fructose ratio of 3 of the suspect samples was ~1:1, while the other had a 1:1.6 ratio. Invert sugar in maple syrups can be produced from sucrose hydrolysis during thermal processing or microbial contamination of the sap [26]. However, the abnormally high invert sugar contents and low sucrose contents in the samples indicate the potential adulteration of maple syrup with inexpensive table syrups. We evaluated commercial table syrup blends that showed similar levels of fructose (13.3 ± 0.8%), glucose (12.3 ± 1.9%), and sucrose 21.7 ± 7.8%) content to the suspect maple syrups.

**Figure 1:**
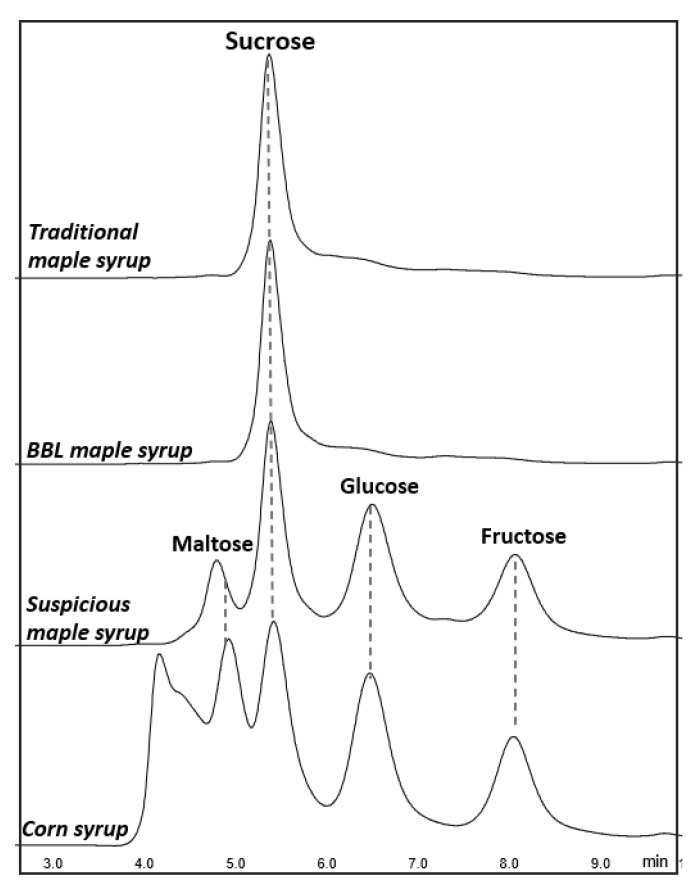
Representative HPLC-RID chromatograms of sugar profiles for traditional maple syrup, BBL maple syrup, suspicious maple syrup and corn syrup.

Figure 1 shows the representative HPLC chromatograms of traditional maple syrup, suspicious maple syrup (which has high invert sugar and low sucrose content), BBL maple syrup and table syrup (specifically corn syrup). The sugar profiles of both traditional and BBL maple syrups obtained by HPLC showed sucrose as the dominant sugar, while the suspicious maple syrup had noticeably high fructose and glucose contents as well as a detectable but low maltose content. In the literature, it has been stated that authentic maple syrup should not have any detectable maltose content [1]. Furthermore, it has been reported that syrup sweeteners, including molasses, high fructose corn syrup, and honey, have wide maltose composition variability, from 3.0–14.4% [28].

The total phenolic contents of traditional maple syrups and BBL maple syrups are summarized in Table 1. In previous reports, total phenolic contents in maple syrups ranged from 200–900 µg/mL, which agreed with our findings [29,30]. Since the FC method is based on the reagent’s chemical reduction, the most problematic assay interference could be the presence of reducing sugars and samples with high protein levels [19]. In traditional maple syrups (except for suspicious ones) and BBL maple syrups, reducing sugars were undetectable. In addition, according to the literature, protein contents in maple syrups are in low concentration (~0–50 ppm) [1]. Therefore, the FC method can be considered a suitable method for analyzing total phenolics in maple syrups. The suspicious maple syrups were excluded from this analysis due to a high-level of reducing sugar (glucose and fructose) content.

The total phenolic content of traditional maple syrups correlated with their color grade and can be separated into golden and amber and dark groups. The dark traditional and BBL maple syrups had significantly higher total phenolic content than the golden and amber maple syrups (*p* < 0.001) according to one-way ANOVA and post hoc LSD tests. Dark maple saps are collected in the later production season when the temperature is warmer (usually at warm springs) and sucrose is converted to invert sugar due to higher microbial activity [1]. Higher invert sugar contents in maple saps result in a stronger Maillard reaction during sap evaporation, giving darker color and stronger flavor in the final maple syrup products. In addition, a higher cultivation temperature of plants and higher activity of beneficial microbe/pathogen/insect feeding increase the total phenolic compounds, which also explain higher phenolics in dark maple syrups [31]. The higher phenolic content of BBL maple syrups could be associated with the aging process in the barrels, resulting in volatile and non-volatile phenolic compounds from the oak wood being absorbed [6] and contributing to their richer, more complex smell and flavor than traditional pure maple syrups.

**Figure 2:**
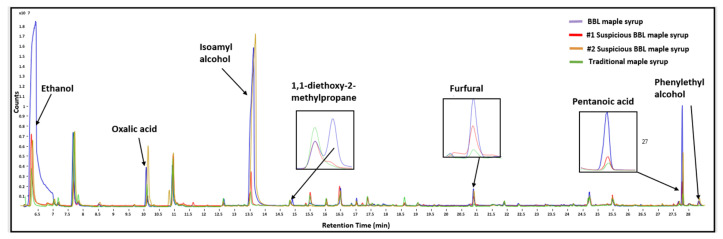
Representative GC-MS chromatograms of volatile compound profiles for BBL maple syrup, suspicious BBL maple syrup and traditional maple syrup.

The unique volatile profile of all maple syrup samples was characterized by GC-MS analysis, and two out of thirteen BBL-aged maple syrups were flagged as having a different volatile profile than the other BBL-aged maple syrup samples. These two samples did not have a different sugar and total phenolic profile than the other BBL samples. As shown in Table A1, a total of 18 volatile compounds were tentatively identified using the NIST 14.L database through a Mass Spectral Library search and were shown to be present in either maple or liquor products. The representative chromatograms for traditional maple syrup, BBL and suspicious BBL maple syrup are shown in Figure 2. There are several noticeable peak differences between BBL and traditional maple syrups. All authentic BBL maple syrups have one unique peak that other traditional and suspicious BBL (*n* = 2) maple syrups do not have, which corresponds to 1,1-diethoxy-2-methylpropane. Previous studies found 1,1-diethoxy-2-methylpropane in aged bourbon whiskey [32]. Therefore, authentic BBL maple syrups could absorb this volatile compound from the bourbon residue in barrels during the long aging process. In addition, one of the suspicious BBL had a similar volatile pattern as traditional samples in that they all had significantly lower contents in ethanol, oxalic acid, isoamyl alcohol, furfural and phenylethyl alcohol than authentic BBL samples, indicating that this suspicious BBL sample might have a minimum or no aging process, or the aging barrel does not contain any bourbon residuals [33]. While the other suspicious BBL sample had a similar volatile pattern as traditional samples, except for contents of isoamyl alcohol and oxalic acid, which were even higher than authentic BBLs, indicating that instead of aging, it might be added with bourbon flavor.

### 3.2. Spectral Information of Maple Syrup Samples

**Figure 3:**
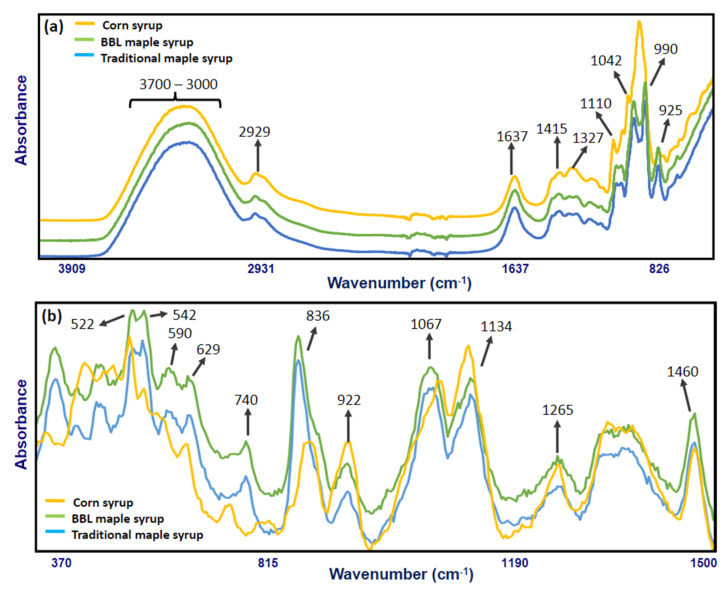
(**a**)FT-IR spectrum band positions and corresponding wavenumbers of traditional maple syrup, BBL maple syrup, and corn syrup at a frequency of 4000–700 cm^−1^ collected using a portable five-reflections ZnSe crystal ATR system. (**b**) Raman spectrum, band positions and corresponding wavenumbers of traditional maple syrup, BBL maple syrup and corn syrup at a frequency of 350–1500 cm^−1^ collected using benchtop Raman with 1064 nm excitation laser.

The characteristic FT-IR absorption spectra of traditional maple syrup, BBL maple syrup, and corn syrup (as an example of table syrups) and their corresponding band assignments for specific functional groups are shown in Figure 3a. Key absorbance signals included the band at 2929 cm^−1^ associated with C-H stretching of the CH2 group in carbohydrates [8]. The band at 1637 cm^−1^ may be mainly related to O-H bonding in water, with minor contributions to C-O stretching in saccharides [34]. The band at 1415 cm^−1^ related to C-H bending [35] and the band at 1327 cm^−1^ related to O-H bending of the C-OH group might attribute to organic acids. The band at 1110 cm^−1^ was associated with C-O stretching of C-O-C linkage, which could be the glycosidic linkage in sucrose. The bands at 1042 and 990 cm^−1^ were associated with C-O stretching in the C-OH group and C-C stretching in carbohydrates, and the band at 927 cm^−1^ was related to C-H stretching [8]. The broadband located around 3600–3000 cm^−1^ was mainly related to O-H bonds stretching in water, which has been reported previously as the major infrared bands of water located at 3490 and 3280 cm^−1^ for O–H stretching [36,37]. The range from 1200 to 800 cm^−1^ could be assigned to the carbohydrates absorption region, mainly related to sucrose, fructose, and glucose absorption bands [8,36].

The characteristic Raman signal of traditional maple syrup, BBL maple syrup, and corn syrup (as an example of table syrups) and their corresponding band assignments for specific functional groups are shown in Figure 3b. The major bands in the Raman spectra were centered in the range of 500–1500 cm^−1^. One major band at 522 cm^−1^ was associated with the deformation of C-C-O and C-C-C [38], while another major band at 542 cm^−1^ is related to an unassigned vibration [8]. The band at 590 cm^−1^ is associated with skeletal vibration [38], and the band at 629 cm^−1^ corresponded with sugar ring deformation [28]. The minor band at 740 cm^−1^ could be due to C-C, C-O stretching in the carbohydrate molecules [13]. The dominant peak at 835 cm^−1^ is responsible for C-C stretching, which is an intense band found in sucrose [39]. The high Raman signal at 835 cm^−1^ band is associated with the high sucrose content (~68%) in maple syrup [4]. Both peaks at 923 and 1067 cm^−1^ are responsible for the combination of vibration C-H bending, especially the C-H bond at C1 position and COH bending [8]. The peak at 1127 cm^−1^ could be due to the deformation of C-O-H, as well as the vibration of C-N, which is found in protein or amino acid [28,38]. The band at 1265 cm^−1^ is associated with the deformation of C-C-H, O-C-H, C-O-H, and the vibration of Amide III, which is a peptide bond, and the band at 1460 cm^−1^ is related to the symmetric deformation in the plane of CH2 [38].

In both FT-IR and Raman spectra, corn syrup was easily differentiated from traditional maple syrup and BBL maple syrup using only visual assessment due to maple syrups’ unique patterns. However, between traditional maple syrup and BBL maple syrup, the spectral differences were not noticeable via visual evaluation due to their similarity. Therefore, a supervised classification method (SIMCA) was used to analyze the spectral data and to determine the class belongings, including traditional maple syrups, BBL maple syrups, and suspicious samples.

### 3.3. Multivariate Data Analysis

#### 3.3.1. SIMCA Classification Model of GC-MS

**Figure A1:**
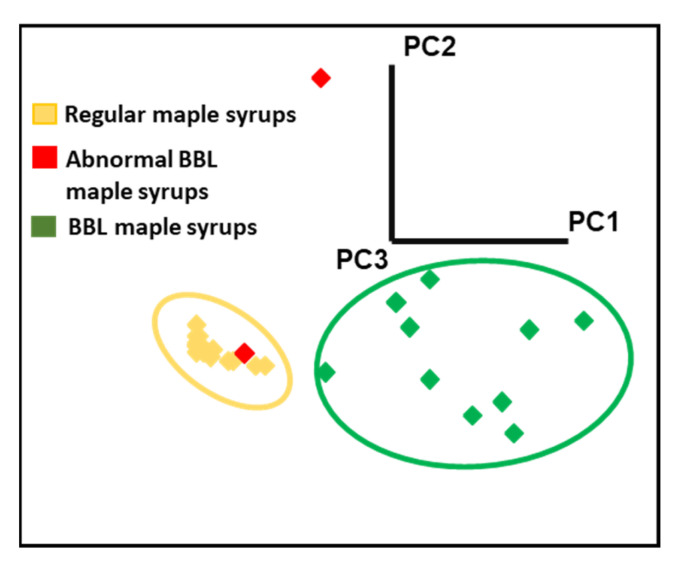
Soft independent modeling of class analogy (SIMCA) projection plots of classification of authentic BBL samples from suspicious BBL and traditional maple syrup samples by GC-MS.

The GC-MS data of volatile compounds in traditional and BBL-aged maple syrup samples were analyzed and grouped using the Soft Independence Modeling of Class Analogy (SIMCA), and the class projection plot is shown in Figure A1. All the authentic BBL maple syrups were successfully discriminated from the traditional maple syrups based on their volatile composition, having an interclass distance (ICD) of 4.1. Furthermore, authentic BBL samples were also successfully differentiated from the suspicious BBL maple syrups (ICD = 2.2), and the classification pattern agreed with the GC-MS data that one of the suspicious BBL grouped with traditional samples, while the other one did not fall into either traditional or authentic sample group. Overall, the five most critical volatile compounds that have the highest impact on SIMCA model discrimination are the order of ethanol, isoamyl alcohol, isobutanol, oxalic acid, and acetoin, which are found to exist in bourbon whiskey or maple sap [33,40]. Therefore, these compounds are significant in authenticating qualified BBL maple syrups from suspicious BBL and traditional maple syrups.

#### 3.3.2. SIMCA Classification Models of FT-IR and Raman Spectroscopy

**Figure 4:**
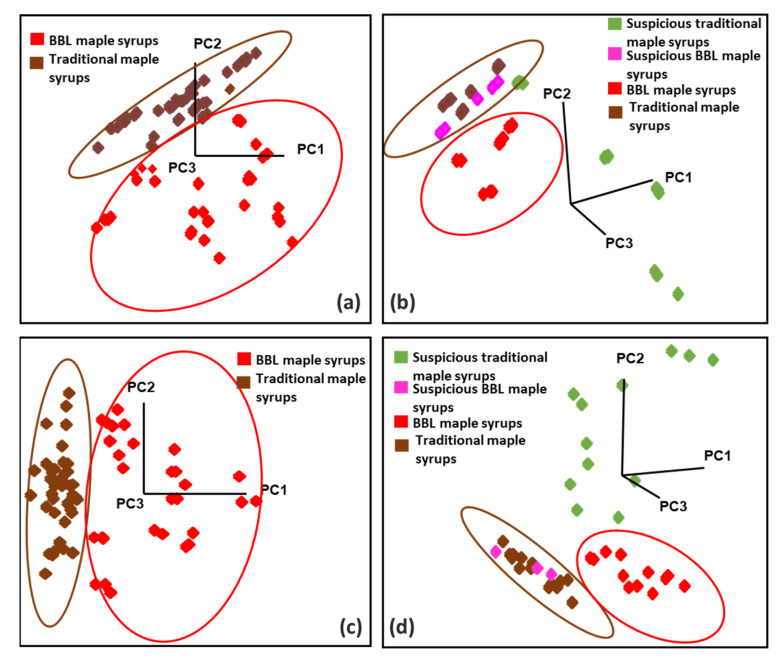
Soft independent modeling of class analogy (SIMCA) projection plots of classification of traditional and BBL maple syrups with (**a**) FT-IR and (**c**) Raman; prediction of external validation sets, including authentic traditional and BBL samples and suspicious samples by (**b**) FT-IR and (**d**) Raman.

Collected FT-IR and Raman spectra were analyzed using SIMCA classification analysis to discriminate traditional and BBL maple syrups from suspicious maple syrups. The multiple-class approach was applied for both FT-IR and Raman spectral data by having two well-established classes existing (BBL and traditional maple syrups) in the training model. The projection plots of training sets are shown in Figure 4a,c. The training sets were developed using 11 BBL maple syrups (two suspicious BBL samples were excluded) and 15 traditional maple syrups (four suspicious traditional samples were excluded). All the BBL maple syrups were assigned to class number 1, and traditional maple syrups were assigned to a different class (#2). Suspicious maple syrups that were found according to the HPLC and GC-MS analysis were assigned as non-target samples and were not represented by the classes. For the FT-IR model, five factors were employed and explained 99.8% of the variances. In the Raman model, six factors were used and explained 98.1% of the variances. In this approach, the training models have ICDs of 4.8 and 2.5, classifying BBL maple syrups into traditional maple syrups based on the FT-IR and Raman methods, respectively.

The SIMCA discriminating power plot interprets variables that have a predominant effect on the sample classification [41]. The fingerprint region of 800–1200 and 800–1000 cm^−1^ was used to discriminate BBL and traditional maple syrups using FT-IR and Raman spectrometers, respectively. For the FT-IR system, most of the model variance was explained by intensity differences of bands located at 878 cm^−1^, which is closely related to the symmetric stretching of the primary alcohol group, and 1034 cm^−1^, which is related to the C-O bond stretching [42,43]. For the Raman system, most of the model variance was explained by the band at 879 cm^−1^, which was also related to the alcohol group’s concentration [44]. Therefore, both FT-IR and Raman methods indicated that differences in compounds with alcoholic groups could explain the variance between BBL and traditional maple syrups. This finding agrees with our GC-MS results since ethanol, isoamyl alcohol, and isobutanol are the top three compounds, assisting BBL maple syrups’ differentiation from traditional maple syrups.

The performances of the supervised multiple-class FT-IR and Raman models were evaluated through an independent external validation set, which comprised four traditional and four BBL maple syrups, four suspicious traditional maple syrups, and two suspicious BBL maple syrups. All four traditional and four BBL maple syrups in the external validation set were tested with all reference analyses, and no abnormal pattern was found. The projection plots of validation sets are shown in Figure 4b, d and displayed well-separated clusters in both methods.

Both FT-IR and Raman models accurately predict all traditional and BBL maple syrups in the correct class (*n* true positive = 8, *n* false negative = 0, sensitivity = 100%), except for two traditional samples with one replication predicted as No Match in the Raman model. In addition, all suspicious traditional maple syrups were predicted as non-pure, and all suspicious BBL samples were predicted as traditional maple syrup, which was consistent with our expectations (*n* false positive = 0, *n* true negative = 6, specificity = 100%). Therefore, all traditional and BBL maple syrups were successfully authenticated by FT-IR and Raman with the multiple-class approach based on their unique chemical composition, and our results agreed with the reference analysis. Our FT-IR and Raman systems displayed a better performance than previous studies of detecting cheap sweetener adulteration in maple syrups, which had 88–100% correctness of discrimination with FT-IR and 98% correctness of discrimination with Raman [8]. Since there is no previous peer-reviewed study investigating BBL maple syrups’ characterization and no formal regulation about the quality control of BBL maple syrups, a larger sample size of BBL maple syrup samples is needed for generating a more comprehensive and representative prediction model in the future.

#### 3.3.3. Regression Models

**Figure A2:**
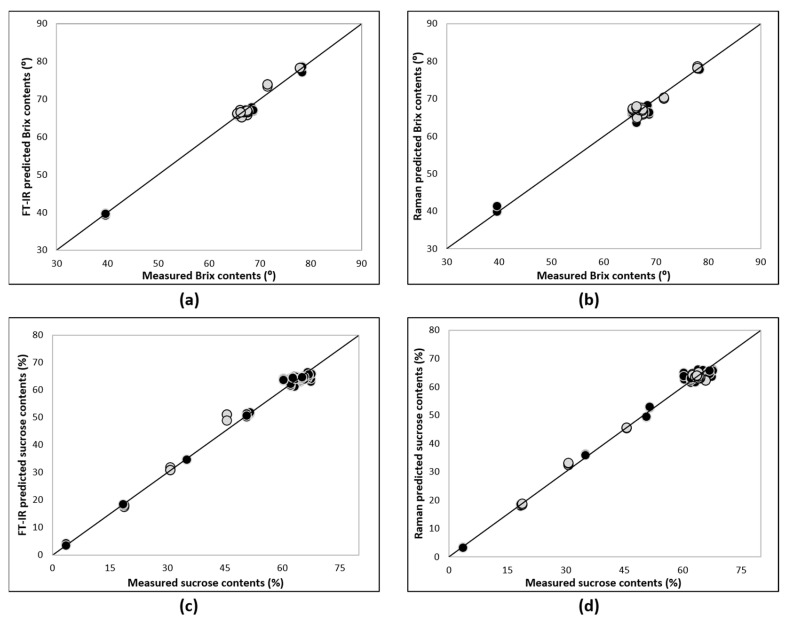
PLSR calibration and validation plots for Brix (**a**,**b**), and sucrose (**c**,**d**) in traditional maple syrups, BBL maple syrups, and table syrup samples utilizing 4500 FT-IR and Raman data, respectively. Black circles represent calibration set samples; gray circles represent external validation set samples.

It is important to monitor the °Brix and sucrose contents in maple syrup to ensure product quality and stability [26]. Partial least square regression (PLSR) prediction models were developed with FT-IR and Raman spectra and reference values of °Brix and sucrose contents (Figure A2). Performance statistics of the PLSR models developed using training (*n* = 26) and external validation (*n* = 11) data sets are listed in Table 2. The number of samples and the range in training models are not all the same due to outlier exclusion. Four and five factors were selected to generate FT-IR and Raman training models, respectively, according to the standard error of cross-validation (SECV) (leave-out-out) result from carrying out the best quality of the models as well as to avoid possible overfitting.

Our PLSR models showed strong correlations (Rcal > 0.98 and Rval > 0.95) in predicting °Brix and sucrose contents in traditional maple syrups, BBL maple syrups, and table syrup samples. The standard error of prediction (SEP) values were 0.88% and 1.66% for FT-IR validation models for °Brix and sucrose, respectively, and were 1.23% and 1.67% for Raman validation models for °Brix and sucrose, respectively. Similar SECV and SEP were obtained, indicating the robustness of the models. Standard errors of laboratory (SEL) for reference methods of °Brix and sucrose were 0.21% and 0.62%, respectively. The SEL values were compared with the prediction performances of the models (SEP), and we found that the SEP values (Table 2) were always higher than those of SEL because the SEP includes not only the sampling and analysis errors but also the spectroscopy and model errors [45]. The SEP obtained for the FT-IR and Raman models were 2.7 times those of the SEL for sucrose, representing good precision of the models [46]. Conversely, the models predicting °Brix had a SEP/SEL ratio of 4.2 (FT-IR) and 5.9 (Raman), which were higher than the SEP/SEL threshold of 2 [46] for acceptable precision compared to the referenced method. However, our models show superior performance compared to reported °Brix predictions for honey using FTIR (R2val = 0.86, SEP = 1.84%) and Raman (R2val = 0.87, SEP = 1.32%) [47,48]. Nickless et al. quantified the total sugar contents in Manuka nectar using FT-IR, reporting Rval = 0.95 and SEP = 1.17% values [15].

**Figure A3:**
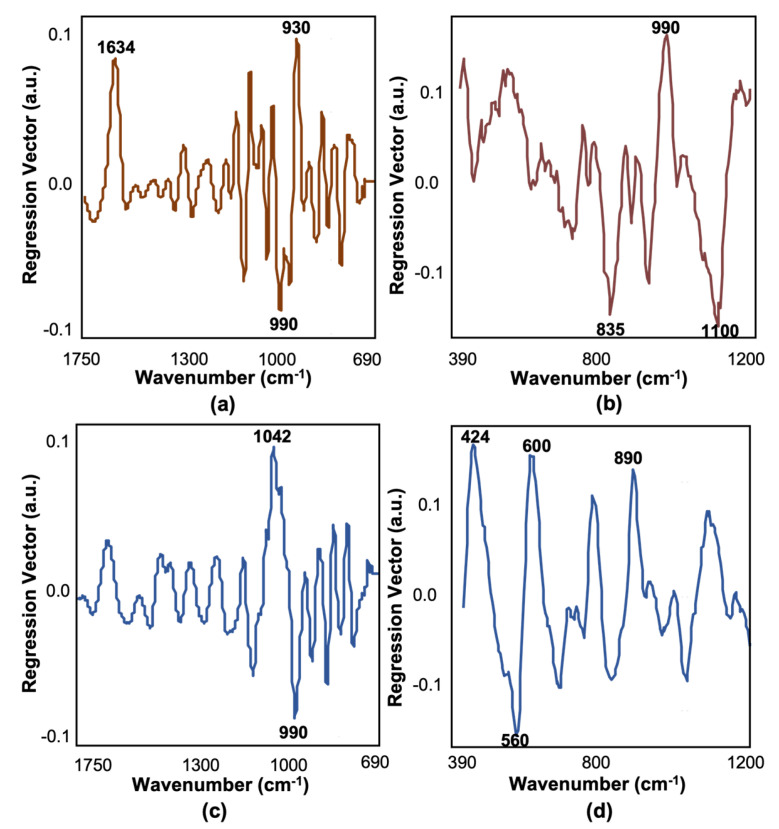
PLSR regression vectors for Brix (**a**,**b**) and sucrose (**c**,**d**), utilizing 4500 FT−IR and Raman data, respectively.

The regression vector plots, shown in Figure A3, help to identify the functional groups whose variance is the highest for correlating between reference values and spectral data. The key FT-IR region for the °Brix and sucrose predictions was in the 1750–700 cm^−1^ range, with distinguished bands centered at 1635 (OH bending vibration characteristic of absorbed water) and the 1125 to 900 cm^−1^ related to C-O stretching and ring vibrational modes of sugars [8,34]. The regression vector plots for Raman data indicated that the bands at 835, 990, 1100 cm^−1^ explained most of the variance for the Brix model, and the bands at 424, 600, and 890 cm^−1^ explained for the Sucrose model. The scattering bands in the vicinity of 424 and 600 cm^−1^ are associated with the deformation of C-C-O and C-C-C [38]. The bands near 990 and 1100 cm^−1^ are related to the deformation modes of saccharides functional groups [28,38].

## 4. Conclusions

In summary, FT-IR and Raman techniques fingerprinted maple syrup products based on their unique chemical composition, allowing for BBL and traditional maple syrup authentication. Both FT-IR and Raman systems combined with SIMCA provided non-destructive, fast, and accurate determination of quality traits in BBL and traditional maple syrups and detected potential maple syrup adulterants. Our results showed that 15% of commercial maple syrup (traditional and/or BBL) samples that were tested and labeled as “pure” exhibited unusual sugar and/or volatile profiles, and both FT-IR and Raman equipment discriminated these suspicious samples from the pure ones. Furthermore, both FT-IR and Raman, combined with PLSR, showed good predictions for all samples’ total °Brix and sucrose contents.

## Figures and Tables

**Table 1:** Reference analysis results of total soluble solids, sugar (sucrose, fructose, and glucose), and total phenolics in traditional, bourbon barrel (BBL)-aged maple syrup and commercial table syrups.

		Traditional Maple Syrup (*n* = 19)		BBL Maple Syrup (*n* = 13)	Table Syrups (*n* = 5)
°**Brix**	Minimum	65.51		65.39	39.63
	Maximum	67.65		68.69	78.27
	Mean	66.57		66.56	67.64
	SD	0.55		0.87	14.32
**Sucrose (%, g/100 g)**	Minimum	22.02		60.13	3.51
	Maximum	67.60		69.42	51.49
	Mean	57.56		63.72	21.75
	SD	14.78		2.73	17.77
**Fructose (%, g/100 g)**	Minimum	0.00		0.00	12.62
	Maximum	17.14		0.00	14.36
	Mean	2.01		0.00	13.31
	SD	4.86		0.00	0.76
**Glucose (%, g/100 g)**	Minimum	0.00		0.00	9.75
	Maximum	17.06		0.00	14.11
	Mean	2.31		0.00	12.34
	SD	5.48		0.00	1.86
		**Golden and Amber** **(** ***n* = 10** **)**	**Dark** **(** ***n*** **= 5)**	**BBL Maple Syrup** **(** ***n*** **= 13)**	**Table Syrups** **(** ***n*** **= 5)**
**Total phenolics** **(µg GAE/mL) ^a^**	Minimum	115.64	387.01	317.37	NA ^c^
	Maximum	338.94	582.39	713.40	NA
	Mean	271.15	479.53	458.25	NA
	SD	64.93	72.85	124.78	NA
	*p*-Value	<0.001 ^b^			NA

^a^ Total phenolics, expressed as micrograms of gallic acid equivalent (GAE) per 1 mL of distilled water. Three unusual maple syrups were excluded from this analysis due to containing of interferences. ^b^ *p* value, based on one-way ANOVA test; there were significant differences in total phenolics between three types of products (*p* < 0.05). Based on post hoc LSD, all samples were significantly different, except for BBL and dark maple syrup (*p* = 0.69). ^c^ Table syrups were excluded from total phenolic analysis.

**Table 2:** Statistics of partial least square regression (PLSR) models developed using a training (*n* = 30) and an external validation (*n* = 7) data set based on FT-IR and Raman spectral data for estimating Brix and sucrose contents in traditional maple syrups, BBL maple syrups, and table syrup samples.

Approach	Sugar	Training Model					External Validation Model			
		Range	*N* ^a^	Factor	SECV ^b^	Rcal	Range	*N* ^c^	SEP ^d^	Rval
**FT-IR**	°**Brix**	39.3–78.7	30	5	0.56	0.99	65.2–78.4	7	0.88	0.98
	**Sucrose**	3.3–66.2	30	4	1.68	0.99	18.4–65.3	7	1.66	0.99
**Raman**	°**Brix**	39.9–78.5	29	5	1.00	0.98	65.0–78.7	7	1.23	0.96
	**Sucrose**	3.5–66.6	30	3	1.69	0.99	17.5–65.1	7	1.67	0.99

^a^ Sample number in the training models. ^b^ Standard error of cross validation. ^c^ Sample number in the external validation models. ^d^ Standard error of prediction.

## Data Availability

The data used to support the findings of this study can be made available by the corresponding author upon request.

## Appendix A

**Table A1:** Summarization of 18 tentatively identified volatile compounds from maple syrup samples utilizing GC-MS.

		2-Methylpropan-1-Ol	Ethanol	3-Hydroxybutan-2-One	1,1-Diethoxy-2-Methylpropane	Pentan-1-ol	2-*O*-Butyl 1-*O*-Propyl Oxalate	3-Methylbut-3-en-1-oll	Unknown Compound	2-(2-Ethylhexoxy)Ethanol	4-Butoxybutan-2-One	2-Methylcyclopent-2-En-1-One	1-*O*-(2-Methylpropyl) 4-*O*-Propan-2-Yl 2,2-Dimethyl-3-Propan-2-Ylbutanedioate	2-Phenylethanol	2-Methylpyrazine	Furan-2-Carbaldehyde	Benzaldehyde	2,6-Dimethylpyrazine	4-Methylbenzaldehyde
	Ions (m/z)	33-43-74	31-45	28-45-88	47-55-103	41-55-70	33-43-74	41-56-68	57-69-89	45-57-71	28-43-73	57-67-96	43-71-159	65-91-122	77-94-105	39-67-96	51-77-106	28-42-108	65-91-119
BBL	min	7.60 × 10^5^	3.10 × 10^7^	2.70 × 10^4^	1.40 × 10^5^	8.10 × 10^6^	5.20 × 10^4^	1.30 × 10^4^	8.70 × 10^2^	2.10 × 10^5^	1.10 × 10^3^	9.20 × 10^3^	1.40 × 10^5^	1.00 × 10^5^	2.40 × 10^4^	5.70 × 10^5^	5.00 × 10^4^	1.40 × 10^4^	4.80 × 10^3^
(*n* = 11)	max	6.10 × 10^6^	8.80 × 10^7^	3.80 × 10^5^	2.70 × 10^6^	3.10 × 10^7^	6.10 × 10^6^	1.60 × 10^5^	3.70 × 10^5^	4.90 × 10^5^	3.20 × 10^5^	1.90 × 10^5^	7.70 × 10^6^	7.20 × 10^5^	2.40 × 10^5^	2.70 × 10^6^	2.10 × 10^5^	1.90 × 10^5^	6.80 × 10^5^
	mean	3.30 × 10^6^	6.10 × 10^7^	1.40 × 10^5^	5.90 × 10^5^	1.90 × 10^7^	3.20 × 10^6^	7.10 × 10^4^	1.40 × 10^5^	3.30 × 10^5^	1.40 × 10^5^	6.60 × 10^4^	4.50 × 10^6^	2.60 × 10^5^	7.60 × 10^4^	1.40 × 10^6^	1.00 × 10^5^	7.20 × 10^4^	3.30 × 10^5^
Abnormal BBL	min	7.60 × 10^5^	8.90 × 10^6^	5.20 × 10^5^	0.00	2.10 × 10^6^	7.60 × 10^5^	1.60 × 10^5^	1.80 × 10^5^	4.20 × 10^5^	6.90 × 10^4^	5.30 × 10^4^	1.80 × 10^5^	2.30 × 10^5^	1.40 × 10^5^	8.00 × 10^5^	7.90 × 10^4^	1.40 × 10^5^	4.10 × 10^5^
(*n* = 2)	max	6.00 × 10^6^	9.40 × 10^6^	8.90 × 10^5^	0.00	3.20 × 10^7^	6.00 × 10^6^	2.70 × 10^5^	2.90 × 10^5^	5.20 × 10^5^	2.20 × 10^5^	2.20 × 10^5^	2.50 × 10^5^	6.00 × 10^5^	2.10 × 10^6^	1.70 × 10^6^	8.80 × 10^4^	1.20 × 10^6^	5.30 × 10^5^
	mean	3.40 × 10^6^	9.10 × 10^6^	7.10 × 10^5^	0.00	1.70 × 10^7^	3.40 × 10^6^	2.10 × 10^5^	2.40 × 10^5^	4.70 × 10^5^	1.40 × 10^5^	1.40 × 10^5^	2.20 × 10^5^	4.20 × 10^5^	1.10 × 10^6^	1.20 × 10^6^	8.40 × 10^4^	6.70 × 10^5^	4.70 × 10^5^
Golden and Amber	min	2.70 × 10^4^	2.90 × 10^5^	6.90 × 10^2^	0.00	9.10 × 10^3^	9.70 × 10^3^	2.60 × 10^3^	5.20 × 10^3^	6.10 × 10^4^	1.30 × 10^3^	4.00 × 10^3^	2.90 × 10^2^	3.20 × 10^3^	8.10 × 10^2^	1.90 × 10^3^	8.10 × 10^2^	1.20 × 10^3^	2.30 × 10^2^
(*n* = 10)	max	1.10 × 10^7^	9.40 × 10^6^	9.70 × 10^5^	0.00	9.80 × 10^5^	2.50 × 10^6^	5.00 × 10^5^	5.80 × 10^5^	7.70 × 10^5^	3.60 × 10^5^	1.50 × 10^5^	3.40 × 10^5^	2.50 × 10^4^	3.70 × 10^5^	5.00 × 10^5^	1.30 × 10^5^	3.80 × 10^5^	8.10 × 10^5^
	mean	2.40 × 10^6^	2.30 × 10^6^	4.90 × 10^5^	0.00	3.30 × 10^5^	8.90 × 10^5^	2.10 × 10^5^	1.90 × 10^5^	3.70 × 10^5^	1.60 × 10^5^	6.30 × 10^4^	1.60 × 10^5^	1.60 × 10^4^	8.80 × 10^4^	1.50 × 10^5^	6.40 × 10^4^	1.10 × 10^5^	3.50 × 10^5^
Dark	min	9.90 × 10^5^	4.30 × 10^6^	6.90 × 10^5^	0.00	2.90 × 10^5^	9.90 × 10^5^	1.80 × 10^5^	1.60 × 10^5^	3.30 × 10^5^	8.20 × 10^4^	6.70 × 10^4^	9.80 × 10^4^	3.00 × 10^4^	2.00 × 10^5^	2.50 × 10^5^	5.80 × 10^4^	4.00 × 10^5^	2.60 × 10^5^
(*n* = 5)	max	2.70 × 10^6^	1.70 × 10^7^	1.00 × 10^6^	0.00	3.00 × 10^6^	2.70 × 10^6^	3.00 × 10^5^	2.20 × 10^5^	5.30 × 10^5^	2.90 × 10^5^	5.30 × 10^5^	2.30 × 10^5^	6.50 × 10^4^	1.60 × 10^6^	5.20 × 10^5^	1.40 × 10^5^	1.30 × 10^6^	4.40 × 10^5^
	mean	1.90 × 10^6^	1.00 × 10^7^	8.80 × 10^5^	0.00	1.50 × 10^6^	1.90 × 10^6^	2.60 × 10^5^	2.00 × 10^5^	4.50 × 10^5^	2.00 × 10^5^	2.20 × 10^5^	1.60 × 10^5^	4.90 × 10^4^	7.00 × 10^5^	3.40 × 10^5^	8.40 × 10^4^	6.40 × 10^5^	3.80 × 10^5^
	*p*-value	<0.001	<0.001	<0.001	<0.001	<0.001	0.002	0.009	0.705	0.403	0.755	0.15	<0.001	<0.001	<0.001	<0.001	0.163	<0.001	0.932
	Ions (m/z)	33-43-74	31-45	28-45-88	47-55-103	41-55-70	33-43-74	41-56-68	57-69-89	45-57-71	28-43-73	57-67-96	43-71-159	65-91-122	77-94-105	39-67-96	51-77-106	28-42-108	65-91-119
BBL	min	7.60 × 10^5^	3.10 × 10^7^	2.70 × 10^4^	1.40 × 10^5^	8.10 × 10^6^	5.20 × 10^4^	1.30 × 10^4^	8.70 × 10^2^	2.10 × 10^5^	1.10 × 10^3^	9.20 × 10^3^	1.40 × 10^5^	1.00 × 10^5^	2.40 × 10^4^	5.70 × 10^5^	5.00 × 10^4^	1.40 × 10^4^	4.80 × 10^3^
(*n* = 11)	max	6.10 × 10^6^	8.80 × 10^7^	3.80 × 10^5^	2.70 × 10^6^	3.10 × 10^7^	6.10 × 10^6^	1.60 × 10^5^	3.70 × 10^5^	4.90 × 10^5^	3.20 × 10^5^	1.90 × 10^5^	7.70 × 10^6^	7.20 × 10^5^	2.40 × 10^5^	2.70 × 10^6^	2.10 × 10^5^	1.90 × 10^5^	6.80 × 10^5^
	mean	3.30 × 10^6^	6.10 × 10^7^	1.40 × 10^5^	5.90 × 10^5^	1.90 × 10^7^	3.20 × 10^6^	7.10 × 10^4^	1.40 × 10^5^	3.30 × 10^5^	1.40 × 10^5^	6.60 × 10^4^	4.50 × 10^6^	2.60 × 10^5^	7.60 × 10^4^	1.40 × 10^6^	1.00 × 10^5^	7.20 × 10^4^	3.30 × 10^5^
Abnormal BBL	min	7.60 × 10^5^	8.90 × 10^6^	5.20 × 10^5^	0.00	2.10 × 10^6^	7.60 × 10^5^	1.60 × 10^5^	1.80 × 10^5^	4.20 × 10^5^	6.90 × 10^4^	5.30 × 10^4^	1.80 × 10^5^	2.30 × 10^5^	1.40 × 10^5^	8.00 × 10^5^	7.90 × 10^4^	1.40 × 10^5^	4.10 × 10^5^
(*n* = 2)	max	6.00 × 10^6^	9.40 × 10^6^	8.90 × 10^5^	0.00	3.20 × 10^7^	6.00 × 10^6^	2.70 × 10^5^	2.90 × 10^5^	5.20 × 10^5^	2.20 × 10^5^	2.20 × 10^5^	2.50 × 10^5^	6.00 × 10^5^	2.10 × 10^6^	1.70 × 10^6^	8.80 × 10^4^	1.20 × 10^6^	5.30 × 10^5^
	mean	3.40 × 10^6^	9.10 × 10^6^	7.10 × 10^5^	0.00	1.70 × 10^7^	3.40 × 10^6^	2.10 × 10^5^	2.40 × 10^5^	4.70 × 10^5^	1.40 × 10^5^	1.40 × 10^5^	2.20 × 10^5^	4.20 × 10^5^	1.10 × 10^6^	1.20 × 10^6^	8.40 × 10^4^	6.70 × 10^5^	4.70 × 10^5^
Golden and Amber	min	2.70 × 10^4^	2.90 × 10^5^	6.90 × 10^2^	0.00	9.10 × 10^3^	9.70 × 10^3^	2.60 × 10^3^	5.20 × 10^3^	6.10 × 10^4^	1.30 × 10^3^	4.00 × 10^3^	2.90 × 10^2^	3.20 × 10^3^	8.10 × 10^2^	1.90 × 10^3^	8.10 × 10^2^	1.20 × 10^3^	2.30 × 10^2^
(*n* = 10)	max	1.10 × 10^7^	9.40 × 10^6^	9.70 × 10^5^	0.00	9.80 × 10^5^	2.50 × 10^6^	5.00 × 10^5^	5.80 × 10^5^	7.70 × 10^5^	3.60 × 10^5^	1.50 × 10^5^	3.40 × 10^5^	2.50 × 10^4^	3.70 × 10^5^	5.00 × 10^5^	1.30 × 10^5^	3.80 × 10^5^	8.10 × 10^5^
	mean	2.40 × 10^6^	2.30 × 10^6^	4.90 × 10^5^	0.00	3.30 × 10^5^	8.90 × 10^5^	2.10 × 10^5^	1.90 × 10^5^	3.70 × 10^5^	1.60 × 10^5^	6.30 × 10^4^	1.60 × 10^5^	1.60 × 10^4^	8.80 × 10^4^	1.50 × 10^5^	6.40 × 10^4^	1.10 × 10^5^	3.50 × 10^5^
Dark	min	9.90 × 10^5^	4.30 × 10^6^	6.90 × 10^5^	0.00	2.90 × 10^5^	9.90 × 10^5^	1.80 × 10^5^	1.60 × 10^5^	3.30 × 10^5^	8.20 × 10^4^	6.70 × 10^4^	9.80 × 10^4^	3.00 × 10^4^	2.00 × 10^5^	2.50 × 10^5^	5.80 × 10^4^	4.00 × 10^5^	2.60 × 10^5^
(*n* = 5)	max	2.70 × 10^6^	1.70 × 10^7^	1.00 × 10^6^	0.00	3.00 × 10^6^	2.70 × 10^6^	3.00 × 10^5^	2.20 × 10^5^	5.30 × 10^5^	2.90 × 10^5^	5.30 × 10^5^	2.30 × 10^5^	6.50 × 10^4^	1.60 × 10^6^	5.20 × 10^5^	1.40 × 10^5^	1.30 × 10^6^	4.40 × 10^5^
	mean	1.90 × 10^6^	1.00 × 10^7^	8.80 × 10^5^	0.00	1.50 × 10^6^	1.90 × 10^6^	2.60 × 10^5^	2.00 × 10^5^	4.50 × 10^5^	2.00 × 10^5^	2.20 × 10^5^	1.60 × 10^5^	4.90 × 10^4^	7.00 × 10^5^	3.40 × 10^5^	8.40 × 10^4^	6.40 × 10^5^	3.80 × 10^5^
	*p*-value	<0.001	<0.001	<0.001	<0.001	<0.001	0.002	0.009	0.705	0.403	0.755	0.15	<0.001	<0.001	<0.001	<0.001	0.163	<0.001	0.932
